# Suicide risk assessment tools and prediction models: new evidence, methodological innovations, outdated criticisms

**DOI:** 10.1136/bmjment-2024-300990

**Published:** 2024-03-14

**Authors:** Aida Seyedsalehi, Seena Fazel

**Affiliations:** 1 Department of Psychiatry, University of Oxford, Oxford, UK; 2 Oxford Health NHS Foundation Trust, Oxford, UK

**Keywords:** Suicide & self-harm, Data Interpretation, Statistical, Machine Learning

## Abstract

The number of prediction models for suicide-related outcomes has grown substantially in recent years. These models aim to assist in stratifying risk, improve clinical decision-making, and facilitate a personalised medicine approach to the prevention of suicidal behaviour. However, there are contrasting views as to whether prediction models have potential to inform and improve assessment of suicide risk. In this perspective, we discuss common misconceptions that characterise criticisms of suicide risk prediction research. First, we discuss the limitations of a classification approach to risk assessment (eg, categorising individuals as low-risk vs high-risk), and highlight the benefits of probability estimation. Second, we argue that the preoccupation with classification measures (such as positive predictive value) when assessing a model’s predictive performance is inappropriate, and discuss the importance of clinical context in determining the most appropriate risk threshold for a given model. Third, we highlight that adequate discriminative ability for a prediction model depends on the clinical area, and emphasise the importance of calibration, which is almost entirely overlooked in the suicide risk prediction literature. Finally, we point out that conclusions about the clinical utility and health-economic value of suicide prediction models should be based on appropriate measures (such as net benefit and decision-analytic modelling), and highlight the role of impact assessment studies. We conclude that the discussion around using suicide prediction models and risk assessment tools requires more nuance and statistical expertise, and that guidelines and suicide prevention strategies should be informed by the new and higher quality evidence in the field.

The growing interest in precision psychiatry in recent years has led to a plethora of risk prediction models, both for the onset of mental illness and for a wide range of course-of-illness outcomes. Predicting the risk of suicide and self-harm has been an area of particular interest. However, there are contrasting views on whether prediction models should be used to assist in suicide risk assessment, with some experts questioning the predictive performance and clinical utility of these models. Here, we discuss four common misconceptions that dominate criticisms of suicide risk assessment tools and prediction models. These have been repeated after the publication in *BMJ Mental Health* of the OxSATS risk calculator,^1^ a novel, scalable and evidence-based approach for estimating 12-month risk of suicide death following self-harm. The OxSATS model was developed in a sample of over 37 000 individuals with hospital presentations of self-harm, using data from Swedish population-based registers. The final 11-item model includes routinely collected sociodemographic and clinical predictors, and showed good discrimination (c-index 0.77, 95% CI 0.75 to 0.78) and calibration (tested by the calibration slope, intercept and calibration plots) in external validation.^1^ To our knowledge, it is the first prediction model in this population that provides probability scores for suicide risk and has been assessed on a full range of performance measures.

The first common misconception among critics of suicide risk prediction, particularly in the UK and Australia,^2 3^ is that all prediction tools invariably have to classify individuals into risk categories (eg, low vs high). This is not the case, and exemplified in one of the most widely advocated prognostic tools in medicine, the Framingham score, which estimates an individual’s probability of developing cardiovascular disease in the next 10 years. In our view, the focus of suicide prediction should shift from classification of individuals (into low-risk vs high-risk groups) to estimating probabilities. Classification implies that all individuals within a risk group should be treated as if they have the same predicted suicide risk. Conversely, two individuals with risk estimates just below and above a classification threshold are assumed to have different levels of risk (and may receive different interventions as a result).^4^ Probability estimates, on the other hand, allow for more personalised decision-making at the individual patient level and hence are more informative.^4^ This is an important distinction between OxSATS (a risk prediction model) and some earlier tools that are classifiers (ie, they do not produce probability estimates).^2 3 5^ In some contexts, guidelines may need to specify a probability threshold for recommending interventions in clinical practice. However, defining risk groups in such contexts still relies on accurate estimation of probabilities.^6^ Furthermore, comparing an individual’s personalised probability estimate with the proposed threshold could improve decision-making in these situations.^4^ An important area for future research is how best to communicate probability estimates in clinical practice to support decision-making around suicide risk management.

Second, arguments against the use of suicide prediction models have largely been based on measures of classification, including sensitivity, specificity and positive predictive value (PPV). However, the overwhelming focus on classification measures when assessing model predictive performance is problematic. While these measures are easily interpretable, their values are strongly dependent on the chosen threshold or cut-off.^4^ There is often no universally optimal threshold for a prediction model, as the choice of threshold should be determined by the clinical context, including the benefits of true positives and the costs of false positive and false negative classifications.^4^ Different clinicians and patients will likely differ in their attitudes towards the costs of misclassification (and therefore risk thresholds for intervention), and any prediction model should be able to accommodate these.^6 7^ For instance, if the intervention involves referral or admission to psychiatric services, the threshold for a given patient may partly be determined by the level of social support (eg, using a higher threshold for referral or admission if the patient has a high degree of social support). Clinicians may also vary in their general propensity to intervene, some having a lower threshold for intervention (ie, more concerned about missing a suicide or self-harm event), while others are more conservative (ie, prioritising avoiding unnecessary interventions).^8^ As such, when evaluating the predictive performance of a model, the primary focus should be on measures of discrimination, such as the area under the receiver operating characteristic curve (AUC), and calibration (ie, the agreement between predicted and observed risks), such as calibration plots. These measures are threshold-independent and assess the quality of predictions across the entire range of model-predicted probabilities.^9^ Ideally, assessment of a model’s performance should also involve examining the (in)stability of its predictions—that is, the extent to which the estimated risks for an individual may differ depending on the particular sample used for model development.^10^ Measures for quantifying model instability at the development stage have recently been proposed by Riley and Collins.^10^ These instability checks can help users decide whether model predictions are likely to be reliable enough in new individuals from the population in which the model was developed.

Third, some critics have suggested that the AUC values for suicide prediction models are too low to be useful.^11^ However, as has been discussed by de Hond *et al*,^12^ the practice of labelling specific AUC values (eg, as poor, moderate, good or excellent) is discouraged as such value judgements are often arbitrary. What is considered ‘good’ discriminative ability for a model depends on the clinical area and on the available alternatives.^13^ While very high AUC values (eg, above 0.90) are sometimes possible in diagnostic prediction modelling (such as the ADNEX model for preoperative diagnosis of ovarian tumours^14^), such values are rare in the context of prognostic prediction. For instance, the most promising models for predicting a range of adverse health outcomes (including mortality) in hospitalised COVID-19 patients, as identified in a recent systematic review,^15^ have AUCs ranging from 0.76 to 0.79. An important related issue which these criticisms fail to recognise is that two models can have similar AUCs despite very different calibration performance. For instance, OxSATS shows reasonably good calibration in external validation,^1^ while some of the first-generation scales^5^ cannot even be assessed on their calibration performance because they do not provide probability estimates. Calibration is a key performance criterion for any model intended to support clinical decision-making, as poorly calibrated risk predictions can be misleading and lead to overtreatment or undertreatment, potentially causing patient harm.^16^ This has been emphasised in numerous methodological and reporting guidelines for prognostic modelling studies,^9 17^ but almost entirely overlooked in the suicide prediction literature.

Fourth, whether or not a model should be used to support clinical decision-making around suicide risk (eg, to support safety planning, screen for more detailed clinical and/or psychosocial assessment or determine treatment) is an empirical question which requires specific measures beyond discrimination and calibration - basing such conclusions on AUC values alone is misguided and involves a conflation of the concepts of model predictive performance and clinical utility. One approach that can be used to evaluate the clinical usefulness of a model for decision-making is to plot the net benefit of the model across a range of clinically reasonable risk thresholds (ie, a decision curve analysis).^18^ As an example, this approach has been recently used to assess the net benefit of a prediction model for violence risk (OxMIV) in a first-episode psychosis population in England.^19^

From a health economics perspective, decision analytical modelling has been used to evaluate the cost-effectiveness of implementing suicide prediction models in different populations and settings. These analyses require a risk threshold to be specified as they reflect the consequences of using the model for decision-making. For instance, it has been shown that implementation of OxMIS^20^—a tool which estimates the probability of suicide in people with severe mental illness—in secondary care in England can lead to cost savings and a small improvement in health outcomes compared with usual care (using a 1% risk threshold to target a high-risk management strategy).^21^ Another economic evaluation study estimated threshold classification accuracy values required for a suicide prediction model to be cost-effective in US primary care.^22^ The analyses showed that for targeting a safety planning and telephone call intervention, at a specificity of 95%, the required PPVs to achieve cost-effectiveness were 0.8% for suicide attempts and 0.07% for suicide deaths. The threshold PPVs were higher for a more resource-intensive intervention (cognitive–behavioural therapy), namely, 1.7% for suicide attempts and 0.2% for suicide deaths.

For low prevalence outcomes like suicide, the PPV of any prediction model, at any given threshold, will be low, and the associated high false positive rate could lead to ‘alarm fatigue’ in clinical practice. However, as highlighted in the study by Ross *et al*,^22^ measures such as PPV and false positive rate cannot be interpreted without considering the clinical context (including the target population, the specific decision that the model is intended to inform, and the relative importance of true vs false positive classifications in that context). This suggests that the same prediction model may have clinical utility and be cost-effective for targeting one particular suicide risk reduction intervention but not another. For instance, if the consequences of being classified as high risk of suicide are not harmful and the target interventions have additional benefits (eg, reducing risk of self-harm or accidental deaths), then a low PPV may not be problematic. Furthermore, there may be specific patient populations (eg, those with a higher prevalence of suicide or non-fatal self-harm) where prediction models are more likely to be clinically useful and/or cost-effective. This further emphasises the point that the most appropriate risk threshold for a given prediction model may be specific to the intervention and population of interest, and should only be determined after the predictive performance of the model (in terms of discrimination and calibration) is thoroughly investigated.^4^

Ultimately, suicide prediction models are only useful in practice if they are linked to effective and scalable interventions,^1^ and if their implementation has a positive impact on clinical decision-making, patient outcomes and cost-effectiveness of care. Quantifying the impact of a prediction model on these outcomes ultimately requires evidence from prospective impact studies (ideally a cluster randomised trial), which are costly and time-consuming.^13^ Such impact studies are rare in prognostic model research, and to our knowledge have not been conducted for any suicide prediction model. However, they are an important step towards implementation for adequately validated models which show evidence of net benefit and the potential for improved patient outcomes and/or favourable cost-effectiveness in decision analytical modelling.^13 23^

In conclusion, we agree with critics of suicide risk prediction that identifying individuals who go on to self-harm or die from suicide is challenging; this is precisely the rationale for developing complex multivariable models using high-quality methods on very large datasets to model risk. There is much to be criticised about the suicide prediction modelling literature; the field must prioritise improved methodological rigour and adherence to best-practice reporting guidelines in the development of new models. There is also a clear need for high-quality external validations in large sample sizes (followed by model updating if necessary), as well as more research assessing the clinical utility and impact of promising models. However, researchers and experts should bring statistical expertise and more nuance in the discussion around using prediction models and risk assessment tools for self-harm and suicide. The field needs to move beyond simplistic blanket statements suggesting that we abandon the endeavour of risk prediction in this area altogether.^1 24^ As discussed here, such statements are not evidence based, do not align with the rest of medicine and come across as ideological. Further, without proper assessment of clinical utility and cost-effectiveness, assertions that PPVs or AUCs of suicide prediction models are too low to be useful should be avoided. Instead, clinical guidelines and suicide prevention strategies should be based on emerging high-quality evidence in the field, and consider a range of issues related to model predictive performance and clinical usefulness.
